# Effects of Five Filamentous Fungi Used in Food Processes on In Vitro and In Vivo Gut Inflammation

**DOI:** 10.3390/jof8090893

**Published:** 2022-08-23

**Authors:** Maxime Poirier, Cindy Hugot, Madeleine Spatz, Gregory Da Costa, Alexia Lapiere, Chloé Michaudel, Camille Danne, Valérie Martin, Philippe Langella, Marie-Laure Michel, Harry Sokol, Patrick Boyaval, Mathias L. Richard

**Affiliations:** 1 Micalis Institute, Université Paris-Saclay, INRAE, AgroParisTech, 78352 Jouy-en-Josas, France; 2 Paris Center for Microbiome Medicine, Fédération Hospitalo-Universitaire, 75012 Paris, France; 3 International Flavors and Fragrances, 20 rue Brunel, 75017 Paris, France; 4 Centre de Recherche Saint-Antoine, Gastroenterology Department, CRSA, AP-HP, Sorbonne Université, INSERM, Saint Antoine Hospital, 75012 Paris, France

**Keywords:** gut microbiota, inflammatory bowel disease, molds

## Abstract

Food processes use different microorganisms, from bacteria to fungi. Yeast strains have been extensively studied, especially *Saccharomyces cerevisiae*. However, to date, very little is known about the potential beneficial effects of molds on gut health as part of gut microbiota. We undertook a comprehensive characterization of five mold strains, *Penicillium* *camemberti*, *P. nalgiovense*, *P. roqueforti*, *Fusarium domesticum*, and *Geotrichum candidum* used in food processes, on their ability to trigger or protect intestinal inflammation using in vitro human cell models and in vivo susceptibility to sodium dextran sulfate-induced colitis. Comparison of spore adhesion to epithelial cells showed a very wide disparity in results, with *F. domesticum* and *P. roqueforti* being the two extremes, with almost no adhesion and 20% adhesion, respectively. Interaction with human immune cells showed mild pro-inflammatory properties of all *Penicillium* strains and no effect of the others. However, the potential anti-inflammatory abilities detected for *G. candidum* in vitro were not confirmed in vivo after oral gavage to mice before and during induced colitis. According to the different series of experiments carried out in this study, the impact of the spores of these molds used in food production is limited, with no specific beneficial or harmful effect on the gut.

## 1. Introduction

Fungi have long been part of the food production processes, particularly the use of yeasts or molds for the production of wine, bread, cheese and of multiple types of fermented food from Europe to Asia [1]. Yeasts that are much easier to handle have been subjected to many researches and some have been genetically engineered for better adaptation to the food processes or even for the addition of new properties [2,3]. Amongst fungi, the most well-known fungus, is the yeast *Saccharomyces cerevisiae* used in wine, beer and bread production [4]. On the contrary, molds, are more difficult to cultivate, they grow slower, and as they often produce often large quantities of spores, they are more difficult to use in the very strict processes of industrial food production [1]. As such, even if they are widely used, especially in cheese production, they have been much less studied than the yeasts. While it has been established that few yeasts strains can have positive effects on the gut, especially for recovery after antibiotic treatments or for irritable bowel syndrome (IBS) [5,6,7], very little has been shown on the potential probiotic effects of molds strains. The main results to date published on potential probiotic effects of filamentous fungi are related to broiler chicks, poultry, and fish production [8,9]. As such, addition of filamentous fungi in the diet may offer different benefits to poultry or fish, including growth promotion, inhibition of pathogen colonization, and improvement of nutrient digestion, as well as enhancement of reproduction. *Acremonium charticola* and *Rhizopus oryzae* isolated from the Indonesian fermented dried cassava showed antibacterial, antifungal, and antioxidant activities, gastrointestinal resilience and fermentative capacity that may be beneficial for the poultry industry [10]. For fish culture, it can be used as source of food and nutrients, Karimi S. et al. showed that it can be a promising alternative for complement of fish food [11]. Additionally, *G. candidum* is tested as a feed additive for fish production to enhance their growth and health status [12,13].

However, no study has been carried out on humans. Consequently, in this project, we aimed at characterizing different molds used daily in the various human food processes. Indeed, as such, these molds are ingested in various quantity by humans and thus are in close contact with the host through diverse interactions with the gut and immune cells. Hence, we characterized their capacities to adhere to these epithelial cells, but we also defined if these epithelial cells trigger any pro- or anti-inflammatory pathways when co-incubated with the mold spores. As the effects of a micro-organism on the gut are very difficult to assess with only in vitro assays, we performed in vivo experiments with five molds: *Penicillium camemberti*, *Penicillium nalgiovense*, *Penicillium roqueforti*, *Fusarium domesticum*, and *Geotrichum candidum*. These five molds were chosen because of their widespread use in food industries in the main production processes of some food products, mainly cheese ripening but also in meat ripening and meat protective micro-flora development [14,15,16]. With the aim of looking for effects on gut in inflammatory settings, we used the five molds in the well described dextran sodium sulfate (DSS)-induced colitis.

## 2. Materials and Methods

### 2.1. Cells Culture

Human enterocyte-like Caco-2 (ATCC, Virginie, USA) and HT29-MTX (Micalis Institut) cells were grown in DMEM (Dulbecco’s Modified Eagle Medium) supplemented with 4.5 g/L of D-Glucose (Sigma-Aldrich, France), pyruvate (Sigma-Aldrich, France), 10% heat-inactivated fetal calf serum (FCS, Eurobio Scientific, France), 50 IU/mL of penicillin (Sigma-Aldrich, France), and 50 ug/mL of streptomycin (Sigma-Aldrich, France) at 37 °C in a 5% CO_2_ atmosphere. Caco-2 required the addition of 1% non-essential amino acids and 1% L-glutamine (Sigma-Aldrich, France). The growth medium was changed every day with fresh medium, and without addition of antibiotics for the last 24 h of incubation prior to performing the adhesion assays.

The HT-29 cell line (passages P10) was obtained from the supplier European Collection of Authenticated Cell Cultures (Sigma Aldrich, France). HT29 cells were cultured in DMEM (GIBCO, Fischer-Scientific France) containing 200 mM of glutamine (GIBCO, Fischer-Scientific, France), 50 IU/mL of penicillin (Sigma-Aldrich, France), 50 μg/mL of streptomycin (Sigma-Aldrich, France), and 10% heat inactivated FCS at 37 °C in a 5% CO_2_ atmosphere. At a confluence of about 80%, HT29 cells were treated with trypsin-EDTA (GIBCO, Fischer-SCI, France) and distributed in 24-well plates (Dutscher, France) with 50,000 cells/well for the following 7 days.

### 2.2. Preparation of Fungi

All strains were supplied by the company International Flavors and Fragrances. *Penicillium camemberti* DGCC5535 (*P. cam*), *P. nalgiovense* DGCC5803 (*P. nal*), *P. roqueforti* DGCC5845 (*P. roq*), *Fusarium domesticum* DGCC3544 (*F. dom*), and *Geotrichum candidum* DGCC3756 (*G. can*) were cultured during 1–2 weeks on Potato-dextrose agar (PDA, BD Difco, Le Pont De Claix, France) at room temperature. The spores were harvested by addition of PBS (GIBCO, Fischer-Scientific, France) with 0.1% Triton X-100 (Sigma-Aldrich, France) and gently scraped. Spores were counted on a Kova™ (KOVA international Inc, USA) slide for concentration evaluation.

### 2.3. Fungal Adhesion on Caco-2 and HT29-MTX cells

Caco-2 and HT29-MTX were seeded at 1.10^5^ cells per well in 12-well plates and were incubated at 37 °C in 5% CO_2_ atmosphere. After two days of culture, the medium was changed every other day until co-incubation. Cells were ready to use 15–21 days after confluence for Caco-2, or 15–18 days after confluence for HT29-MTX when they start to produce mucus. For co-incubation, 2.10^6^ spores were used per each well.

#### 2.3.1. Quantitative Assessment of the Adhesion

The adherence of filamentous strains to cultured enterocytes was assayed by RT-qPCR method. After 1 h of co-incubation (37 °C, 5% CO_2_) enterocytes, were washed five times with 0.5 mL of PBS (GIBCO, Fischer-Scientific, France), then scraped in 0.5 mL of solution of Tris/HCL 50 mM pH = 7.5 (Sigma-Aldrich, France) and EDTA 20 mM (Sigma-Aldrich, France) and collected in 2-mL screw tubes already containing zirconia/silica beads (half 0.5 mm/ half 0.1 mm). Charge control wells (adhesion 100%), were scraped directly in the medium without washing, collected in 2-mL tubes and centrifuged for 10 min at max speed (14,000× *g*). The percentage of adhesion represent the percentage of cells that can be recovered after 1 h adhesion period and five washes compared to the total cells initially deposited on the cell lines (charge control wells).

#### 2.3.2. DNA Extraction

DNA extraction was done using the “Querol protocol” as described in the following reference [17]. Quality and concentration of DNA were checked using a NanoDrop apparatus (Thermo Fisher Scientific, USA). qPCR was performed with 1.5 µg of DNA and using a Luna^®^ Universal qPCR Master Mix (New England Biolabs, USA) in a StepOnePlus apparatus (Applied Biosystems, Foster City, USA) with specific fungi oligonucleotides (TEF1a). The primer sequences of the amplified target are listed in Table 1.

### 2.4. IL-8 Production by HT29 after Co-Culture with Fungi

Before HT29 stimulation, the cells were cultured for 24 h in a medium containing only 5% FCS. For stimulation, TNF-α (PEPROTECH, France) was added at 5 ng/mL. Co-incubation with fungi was realized by adding a yeast suspension with a multiplicity of infection (MOI) of 5 in triplicate, and the plates were incubated for 6 h at 37 °C, 5% CO_2_.

After treatment, the culture supernatants were collected for the assay. IL-8 quantification was performed using an IL-8 ELISA kit (BIOLEGEND, France) and following the protocol provided by the provider.

### 2.5. Human Peripheral Blood Mononuclear Cells (PBMCs) and Fungi Co-Incubation

Human PBMCs were purified from whole blood (Etablissement Français du Sang, Le Chesnay, FRANCE) using Histopaque-1077 (Sigma Aldrich, France) gradient centrifugation. PBMCs were harvested from the interface, washed three times with sterile PBS (GIBCO, Fischer-Scientific, France), and diluted in 5 mL (for 10 mL of blood) of RPMI 1640 (GIBCO, Fischer-Scientific, France) containing 10% SCF (Eurobio Scientific) and 1% penicillin/streptomycin (Sigma-Aldrich, France). PBMC concentration was determined by using Kova slide (Kova international, USA) and adjusted to a final concentration of 1 × 10^6^/mL.

One hundred microliters at 1 × 10^6^/mL of freshly isolated PBMCs were seeded into a 96-well culture plate (U-bottom), and 100 µL at 5 × 10^6^/mL of fungi suspension was added to each well (triplicate) for a final ratio at 1 (PMBC): 5 (Fungi). Lipopolysaccharides (LPS; Invivogen) at final concentration to 10 ng/mL were used as the positive control treatment. To determine cytokine expression, samples were incubated for 24 h at 37 °C and 5% CO_2_ and the supernatant was collected for ELISA assays.

### 2.6. Colitis Model in the Mice

Eight-week-old female C57BL/6J mice were purchased from Janvier Laboratory (Le Genest, France) and used 1 week after reception. Animals were kept in humidity- and temperature-controlled rooms under a 12 h light-dark cycle and had access to a chow diet and water ad libitum. All experiments were performed in accordance with the ethics committee “Comite d’Ethique en Experimentation Animale” (COMETHEA C2EA—45, Jouy en Josas, France). Every experiment was repeated at least two times (n = 10).

Prior to DSS administration, the mice were gavaged with a suspension of fungi: 1.10^7^ spores per gavage/mouse/day.

One week after starting the fungal administration, mice were given 2% (weight/volume) colitis grade DSS (molecular weight, 36,000–50,000; MP Biomedicals, Solon, OH, USA) dissolved in the drinking water *ad libitum* for 7 days, followed by a recovery period (water only) of 5 days. Animals were monitored daily for weight loss and disease activity index (DAI). The DAI is described in Table 2 and includes three parameters with a score notation from 1 to 4: weight loss, stool consistency, and presence of blood in the feces.

### 2.7. Tissues and Samples

Mice were euthanized by cervical dislocation. The proximal colon was flushed and frozen for further RNA extraction. Fecal samples were collected at Day 7 and at the end of the protocol (Day 12) and frozen for fecal lipocalin level measurements. All samples were stored at −80 °C until use.

### 2.8. Quantification of Fecal Lipocalin (LCN2) Levels

Lipocalin quantification by ELISA function as a non-invasive, sensitive, dynamic, stable, and cost-effective means to monitor intestinal inflammation in mice [18]. Frozen fecal samples were weighed and suspended in cold PBS (GIBCO, Fischer-Scientific, France). The samples were then agitated on a Precellys (Bertin Corp., France) for 40 s at 5000 rpm with 4.5 mm glass beads to obtain a homogenous fecal suspension. The samples were then centrifuged for 5 min at 10,000× *g* (4 °C), and clear supernatants were collected and stored at −20°C until analysis. LCN2 levels were estimated using a DuoSet murine LCN2 ELISA kit (R&D Systems, USA) according to the manufacturer’s instructions and expressed as pg/mg of stool.

### 2.9. RNA Extraction and Gene Expression Analysis Using Quantitative Real-Time PCR (qRT–PCR)

Total RNA was isolated from colon samples using an RNeasy Mini Kit (Qiagen, Hilden, Germany), including a DNAse treatment step, according to the manufacturer’s instructions. Quality and concentration of RNA were checked using a NanoDrop apparatus (Thermo Fisher Scientific, USA). RT–PCR was performed using a LunaScript RT SuperMix Kit (New England Biolabs, USA) followed by qPCR using Luna^®^ Universal qPCR Master Mix (New England Biolabs, USA) in a StepOnePlus apparatus (Applied Biosystems, Foster City, USA) with specific mouse oligonucleotides. Amplification was initiated with an enzyme activation step at 95 °C for 10 min, followed by 40 cycles consisting of a 15 s denaturation step at 95 °C and a 60 s annealing step at 60 °C and a melting curve consisting of a step of temperature increase from 60 to 95 °C with a fluorescence analysis every 0.3 s. The primer sequences of the amplified target are listed in Table 1. We used the 2^−ΔΔCt^ quantification method with mouse GAPDH as a control.

### 2.10. Statistical Analysis

GraphPad Prism version 7 (San Diego, USA) was used for all analyses and preparation of graphs. For all data displayed in graphs, the results are expressed as the mean ± SEM (n = 22 to 25 per group). For comparisons among more than two groups, one-way analysis of variance (ANOVA) and a post hoc Tukey test or a nonparametric Kruskal–Wallis test followed by a post hoc Dunn’s test were used. For comparisons with multiple factors, two-way ANOVA and a post hoc Tukey test were used. For all statistical tests, differences with a p value less than 0.05 were considered to be statistically significant: * *p* < 0.05, ** *p* < 0.01, *** *p* < 0.001.

## 3. Results

### 3.1. Adhesion Properties Strongly Vary between Filamentous Strains

**Figure 1:**
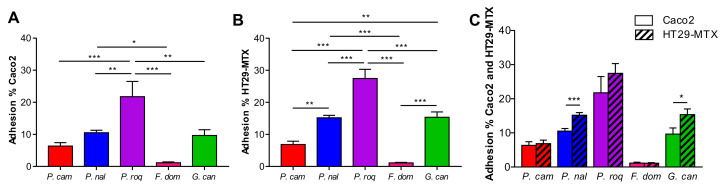
Adhesion properties between filamentous strains. Adherence capacity of *Penicillium camemberti* (*P. cam*), *P. nalgiovense* (*P. nal*), *P. roqueforti* (*P. roq*), *Fusarium domesticum* (*F. dom*), or *Geotrichum candidum* (*G. can*) on (**A**) Caco-2, (**B**) HT29-MTX, and (**C**) both cell lines. * *p* < 0.05, ** *p* < 0.01, *** *p* < 0.001.

The capacity to interact with the host and to persist in the gut might be partially due to the capacity of the spores to adhere to gut surfaces. The gut epithelium is composed of different type of cells (enterocytes, goblet cells, Paneth cells, etc.) covered by a layer of mucus produced mainly but not only by the goblet cells [19]. To identify the capacity to adhere to the cells or more specifically to the mucus, we performed an adhesion assay in vitro on two types of epithelial cell lines: Cac-o2 and HT29-MTX cells. Both are human cells with different properties: Caco-2 are absorptive enterocytes, while HT29-MTX are mucus secretive cells [20]. We chose to compare these two cell types so we could describe how the mucus may affect fungus-to-cell adhesion. In Figure 1, we observed that the capacity of adhesion on Caco-2 cells ranged from 2% to more than 20% (Figure 1A) and that it ranged on HT29-MTX from 2% to 30% (Figure 1B). Interestingly, the level of adhesion was very variable between strains, with *P. roqueforti* being the most adherent strain, while *F. domesticum* was the least. However, the pattern of adhesion, although somewhat variable in terms of absolute values, was very similar between Caco-2 and HT29-MTX cells. Indeed *P. roqueforti* and *F. domesticum* were again the two extremes (Figure 1C).

Altogether, these data show that presence of mucus do not drastically modify the adherence capacities of the spores since the global patterns are comparable between the two cell lines. However, mucus seems to slightly increase the adhesion for 2 strains that adhere significantly more on HT29-MTX than on Caco-2 cell (*P. nalgiovense* and *G. candidum*), *F. domesticum* is completely non adherent on both models, and *P. roqueforti* is very adherent on both models, while *P. camemberti*’s poor adhesion properties are not influenced by the presence of mucus (Figure 1C).

### 3.2. G. candidum Spores Elicite Low Inflammation on HT29 Epithelial Cells

**Figure 2:**
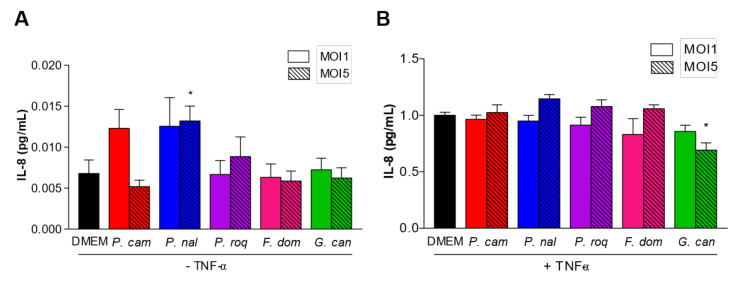
IL-8 production between filamentous strains. IL-8 production by HT29 after co-culture with filamentous strains *Penicillium camemberti* (*P. cam*), *P. nalgiovense* (*P. nal*), *P. roqueforti* (*P. roq*), *Fusarium domesticum* (*F. dom*), or *Geotrichum candidum* (*G. can*) at MOI1 and MOI5, (**A**) without or (**B**) with TNF-α priming. For statistical comparisons, (*) indicates versus DMEM. * *p* < 0.05.

HT29 are epithelial cells. As such, they are at the interface between the host and the microbiota, and they have developed the capacity to produce different type of cytokines to communicate with the immune cells at the periphery (*lamina propria*) and regulate the immune system response [21]. Many laboratories have thus chosen HT29 to characterize the potential pro- or anti-inflammatory effect of molecules or micro-organisms. One classical assay is to follow the levels of IL-8 produced by HT29 after TNF-α induction, and the effect of the addition of a molecule or a micro-organism [22]. To determine whether the spores could trigger a pro-inflammatory reaction when recognized by the epithelial cells, we co-incubated HT29 cells without TNF-α priming. Co-incubation of the five fungal strains with HT29 cells for 6 h induced the production of very low levels of IL-8 in most of the strains, except for *P. camemberti* and *P. nalgiovense* (Figure 2A), while TNF- α induced normal levels of IL-8 (Figure 2B), suggesting that the spores of the different fungi were not recognized by HT29′s pathogen-associated molecular patterns (PAMPs) at least at MOI1 or MOI5.

To show the potential anti-inflammatory effect of the fungal strains, we co-incubated HT29 primed with TNF-α with spores at MOI1 and MOI5 (Figure 2B). Interestingly, co-incubation of HT29 primed with TNF-α with spores at MOI5 showed a tendency of increased level of IL-8 production for all strains but *G. candidum*. At this concentration, *G. candidum* spores significantly reduced the IL-8 production compared to all the other strains, suggesting an anti-inflammatory effect of these spores on epithelial cells.

### 3.3. Human Immune Cells Differentially Recognize Fungal Spores

**Figure 3:**
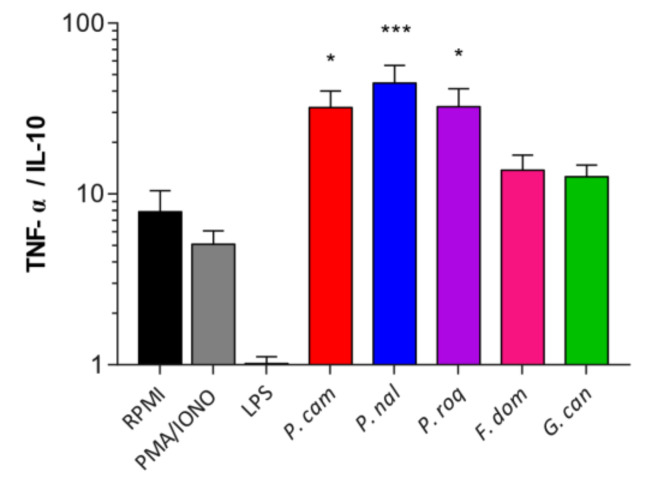
Human immune cells differentially recognize fungal spores. Ratio of TNF-α/IL-10 production of the culture supernatants of peripheral blood mononuclear cells (PBMCs) co-incubated with *Penicillium camemberti* (*P. cam*), *P. nalgiovense* (*P. nal*), *P. roqueforti* (*P. roq*), *Fusarium domesticum* (*F. dom*), or *Geotrichum candidum* (*G. can*) for 24 h at MOI5. For statistical comparisons, (*) indicates versus RPMI. * *p* < 0.05, *** *p* < 0.001.

In addition to the recognition and effect of spores on epithelial cells, in order to have a full image of the effect of these spores when interacting with their host, we compared how they are detected by human immune cells using peripheral blood mononuclear cells (PBMCs). PBMCs were subjected to 24 h of co-incubation with spores at MOI5 [23]. ELISA was conducted on the culture supernatants and IL-10 and TNF-α production quantified and represented as a ratio of TNF-α/IL-10. The results showed that *F. domesticum* and *G. candidum* do not trigger any specific pro- or anti-inflammatory effects on the PBMCs with comparable results compared to the control (Figure 3). However, *P. camemberti*, *P. nalgiovense,* and *P. roqueforti* show a significant pro-inflammatory effect. These data suggest that some epitopes present at the spore surface of the genus *Penicillium* are recognized by the PAMPs present on the PBMCs, thus activating the immune response pathway into a defense mechanism.

### 3.4. DSS-Induced Colitis Is Not Influenced by Fungal Spores’ Oral Administration

**Figure 4:**
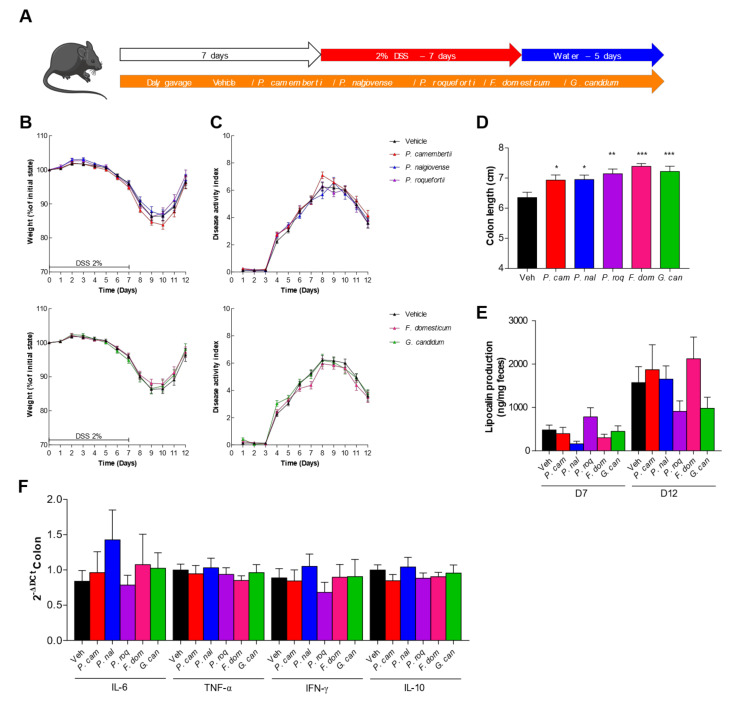
Dextran sodium sulfate (DSS)-induced colitis is not influenced by fungal spores’ colonization. (**A**–**F**) Mice received vehicle (Veh, PBS), *Penicillium camemberti* (*P. cam*), *P. nalgiovense* (*P. nal*), *P. roqueforti* (*P. roq*), *Fusarium domesticum* (*F. dom*), or *Geotrichum candidum* (*G. can*) for 7 days and then dextran sulfate sodium (DSS) for 7 days. Vehicle n = 23, *P. cam* n = 25, *P. nal* n = 22, *P. roq* n = 24, *F. dom* n = 26, *G. can* n = 24. A. Experimental design for the administration of DSS. B. Weight of DSS-exposed mice. C. Disease activity index (DAI) of DSS-exposed mice. D. Length of the colons of mice treated with DSS. E. Intestinal inflammation, expressed as the lipocalin levels in feces at Day 7 and Day 12. F. Intestinal cytokines IL-6, TNF-α, IFN-γ, and IL-10 in the colon (qPCR). For statistical comparisons, (*) indicates versus vehicle. * *p* < 0.05, ** *p* < 0.01, *** *p* < 0.001.

In vitro data have shown diverse effects of the spore on host cells. With the aim to have a complete characterization of the potential effect of these spores as probiotic, especially *G. candidum,* identified on the HT29 in vitro test, we tested them in a more complex model in vivo. We used the DSS-induced colitis that is known to mimic the symptoms of ulcerative colitis in humans, with loss of weight, presence of blood in the feces, and diarrhea. We thus tested the effect of our fungal spores on this model by pre-treating the mice with a daily gavage of spores for 1 week before beginning the DSS treatment (Figure 4A). The mice gavaged with 10^7^ spores per day did not show any protective or worsening effect during DSS treatment when monitoring the weight curves (Figure 4B) or the disease activity index (DAI) (Figure 4C). The measure of the colon length, another macroscopic marker of colitis, did not show any significant change dependent on the treatment (Figure 4D). Similarly, quantification of lipocalin, a fecal marker of intestinal inflammation [18], (Figure 4E) and colonic expression of the proinflammatory cytokine (Figure 4F) showed little variation but without any strong induction compared to the control, suggesting no specific effect of the fungi in this model. Interestingly, *P. roqueforti* and *G. candidum* treatment elicited a lower, although not significant, quantity of lipocalin detected in the feces, suggesting a potential positive effect.

## 4. Discussion

While data on yeasts slowly accumulated in the last decade, the description and characterization of molds was much slower. This difference was mostly due to practical reasons. Molds are more difficult to handle in the laboratory and very few genetic tools have been developed [24]. However, these fungal strains have been used in food processes since ages and the need for comprehensive analysis of these microorganisms is increasing [25]. Filamentous fungi are the source of dietary proteins, lipids and fatty acids, vitamins, fiber, and flavors, and can improve the organoleptic properties of processed foods (cheese or meat). In addition, they are often key ingredients in nutritional or therapeutic supplements because of the diversity of metabolites produced. Mycelia can also improve the efficiency of feed conversion, intestinal health, and well-being of livestock [24]. Indeed, the role of the gut microbiota in health and disease is now recognized by the scientific and medical community, and the specific role of the fungal part of the microbiota is descried in several diseases [26]. In this project, we choose to describe five different strains of mold used in the food industry: *Penicillium camemberti*, *P. nalgiovense*, *P. roqueforti*, *Fusarium domesticum,* and *Geotrichum candidum*. These strains were selected mainly because these are very common strains used in well-known cheese or meat productions, especially in France where there is a very old tradition of cheese and meat ripening. *P. camemberti*, *G. candidum,* and *F. domesticum* are mostly used for cheese crust production and cheese ripening of the Camembert, St Nectaire, or Reblochon [27,28], while *P. roqueforti* is part of the Roquefort cheese development and participate to the aroma development during ripening [29,30,31,32]). *P. nalgiovense* composed the starter culture of dry fermented sausages and the protective flora used during ham and sausage production, a microbiota that develop at the surface and block the overgrowth of potential pathobiont [33,34]. Interestingly, *P. camemberti* in addition to its role in cheese aroma production has been described in the microbiota developed at the surface to dry meat and is used in the production of pork dry sausages like *P. nalgiovense* [35,36]. As such, they are ingested in large quantities in their filamentous or spore forms and can influence the microbial equilibrium in the gut.

Using various in vitro assays, we characterized how spores of these molds interact with diverse host cells and also if their presence in the gut influence positively or negatively in a in vivo inflammatory setup. *P. nalgiovense* showed a tendency to have inflammatory effect on several tests, but with low level of induction even if in some cases we reached statistical significance. As such, it might be interesting to initiate further studies aiming at a better characterization of these effects and thus dismiss any deleterious effect of this strain on gut health.

Interestingly *G. candidum* spores were identified as having an anti-inflammatory effect on HT29 epithelial cells with clear reduction of IL-8 production. However, even if the level of lipocalin after the recovery was lower, no additional statistically significant effect in vitro or in vivo were shown when we used *G. candidum*, suggesting a mild effect of these spores. This result will be important to follow in order to understand the pathway implicated in this reduction of inflammation and the effectors involved. Further experiments are needed in order to tackle these questions using specific pathway inhibitors or KO cell lines. The use of filaments or germlings could also be an alternative for following works. Knowing that this strain shows a positive effect on fish growth and immune response [12] and those numerous filamentous fungi have been used in food supplement with global health positive effects [37,38] it might be interesting to continue in this direction and initiate tests of administration of some filamentous fungi in the food, monitoring the effect on global health modification.

## 5. Conclusions

Consequently, according to the various sets of experiments realized in this project, we can consider that the impact of spores from these specific molds used in food production is limited; however, this study is restricted to the use of spores and various literature data suggests that mycelia could be very important. Indeed, these elongated cells have a strong metabolic activity with the secretion of many types of molecules. The effect of these molecules on the gut microbiota and the host health are surely interesting to describe in following works and might elicit much stronger effects.

## Figures and Tables

**Table 1:** Primer sequences of the amplified target.

Name	5′–Forward–3′	5′–Reverse–3′
GAPDH	AACTTTGGCATTGTGGAAGG	ACACATTGGGGGTAGGAACA
IL-10	AGAAGCATGGCCCAGAAATCA	GGCCTTGTAGACACCTTGGT
IL-6	GTAGCTATGGTACTCCAGAAGAC	ACGATGATGCACTTGCAGAA
INF-γ	CCATCCTTTTGCCAGTTCCTC	ATGAACGCTACACACTGCATC
TNF-α	GACCCTCACACTCAGATCATCTTCT	CCACTTGGTGGTTTGCTACGA
TEF1a	GATTTCATCAAGAACATGAT	GACGTTGAAACCGACGTTGTC

**Table 2:** Disease activity index calculation table.

Score	Consistency Stool	Blood
0	Normal	Negative (−)
2	Loose stools	+
4	Diarrhea	Bleeding

## Data Availability

The data presented in this study is contained within the article.
